# Supplementary material for: Non-coding cis-element of Period2 is essential for maintaining organismal circadian behaviour and body temperature rhythmicity
Source: Nat Commun. 2019 Jun 12;10:2563. doi: 10.1038/s41467-019-10532-2 (PMC6561950; doi:10.1038/s41467-019-10532-2)
Supplement: Supplementary file 1 — Supplementary Information [file 41467_2019_10532_MOESM1_ESM.pdf]

Supplementary Information

Non-coding *cis*-element of *Period2* is essential for maintaining organismal circadian behaviour and body temperature rhythmicity

Doi, Okamura et al.

Supplementary Figure 1

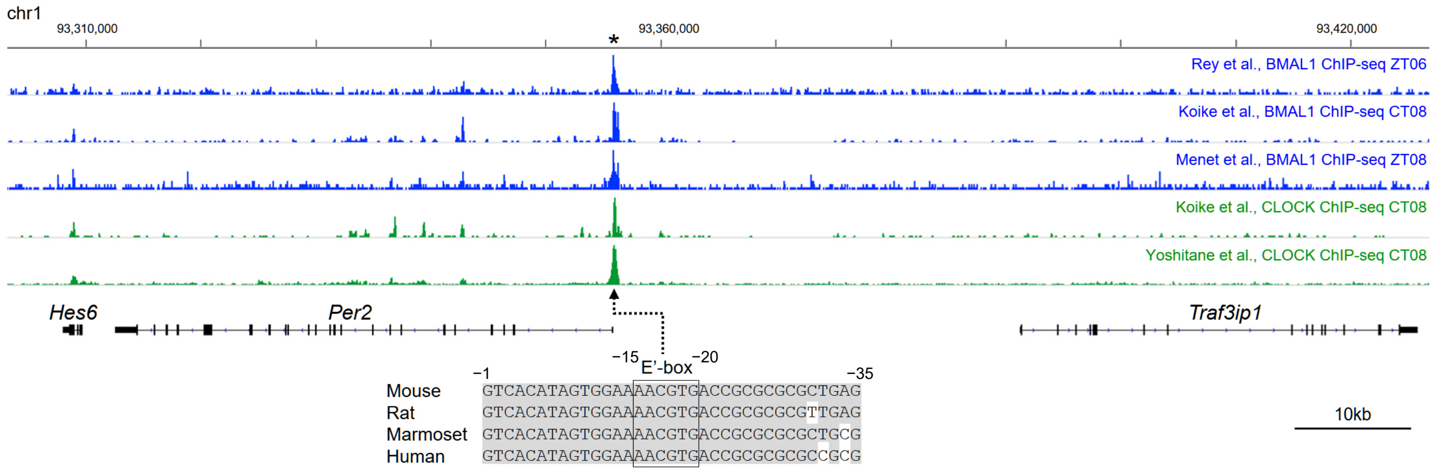

Supplementary Figure 1 | Genome-wide ChIP-seq database analyses map the predominant peak of the clock-protein-binding activity around the E′-box in the vicinity of the transcription start site of the *Per2*.

Blue: BMAL1 ChIP-seq data reported by Rey *et al*. PLoS Biol, 2011 (SRA ID: SRX038434), Koike *et al*. Science, 2012 (SRX174621), and Menet *et al*. Elife, 2012 (SRX131983).

Green: CLOCK ChIP-seq data reported by Koike *et al*. Science, 2012 (SRX174644) and Yoshitane *et al*. Mol Cell Biol, 2014 (DRX011954).

In all reports, mouse livers were analyzed. The data for either ZT06, ZT08, or CT08 are shown.

BigWig files corresponding to each ChIP-seq experiment were downloaded from either the Gene Expression Omnibus (https://www.ncbi.nlm.nih.gov/geo/) or the ChIP-Atlas database (http://chip-atlas.org/) and visualized with the Integrative Genomics Viewer (http://software.broadinstitute.org/software/igv/).

Arrow indicates the position of the *Per2* E′-box, the sequence of which is conserved among mouse, rat, marmoset, and human. Numbers on the alignment indicate the position relative to the mouse *Per2* transcription start site.

Supplementary Figure 2

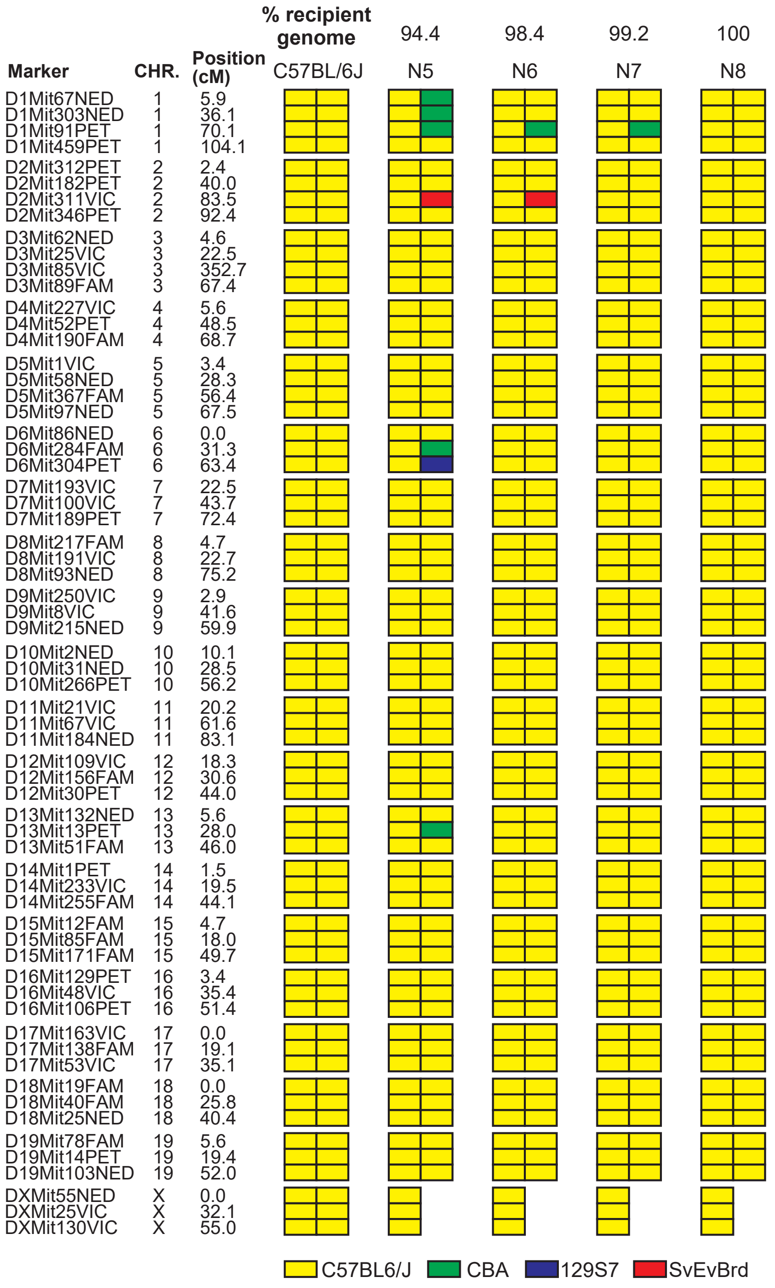

Supplementary Figure 2 | Marker-assisted backcrossing of the *Per2* E′-box (−20/−15) mutant mice to C57BL/6J background. Genotypes of 64 microsatellite markers are color-coded: yellow, C57BL/6J; green, CBA (derived from TT2 ES cell); blue, 129S7 (derived from ROSA26-PBase mice); and red, SvEvBrd (derived from ROSA26-PBase mice). Backcross generations (N5 to N8) and deduced percentages of recipient genome are indicated at the top of each column.

Supplementary Figure 3

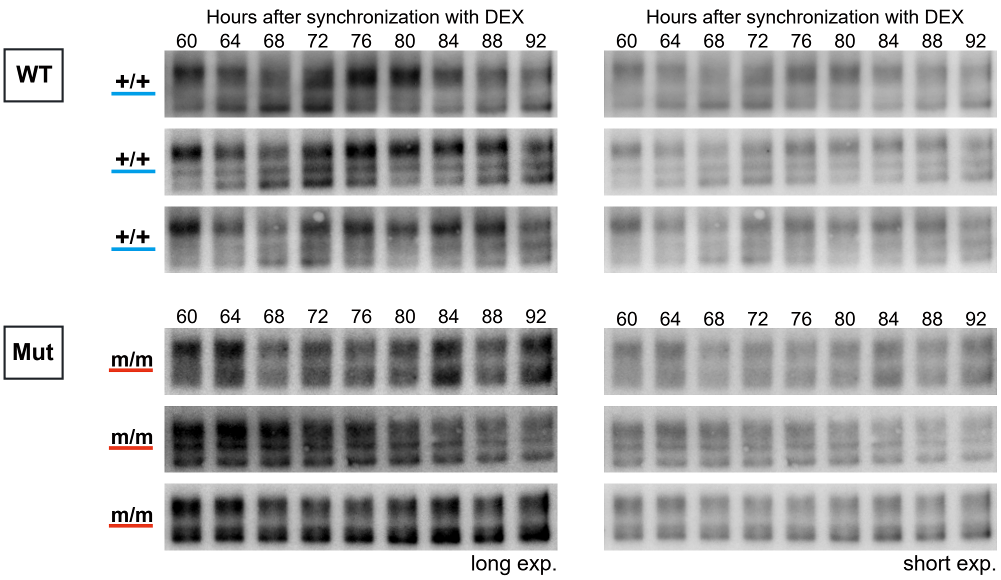

Supplementary Figure 3 (related to Fig. 2a) | Immunoblots showing temporal profiles of PER2 protein expression in *Per2*E′$^{+/+}$ and *Per2*E′$^{m/m}$ fibroblasts. Cells were synchronized with DEX as depicted in **Fig. 2a** and sampled every 4 h after 60 h. Two exposures (long and short) of the same sets of protein blot data from *Per2*E′$^{+/+}$ (n = 3) and *Per2*E′$^{m/m}$ (n = 3) are shown. Source data are provided as a Source Data file. Note that PER2 proteins in the mutant cells appeared relatively variable or not circadian, compared to those in WT cells.

Supplementary Figure 4

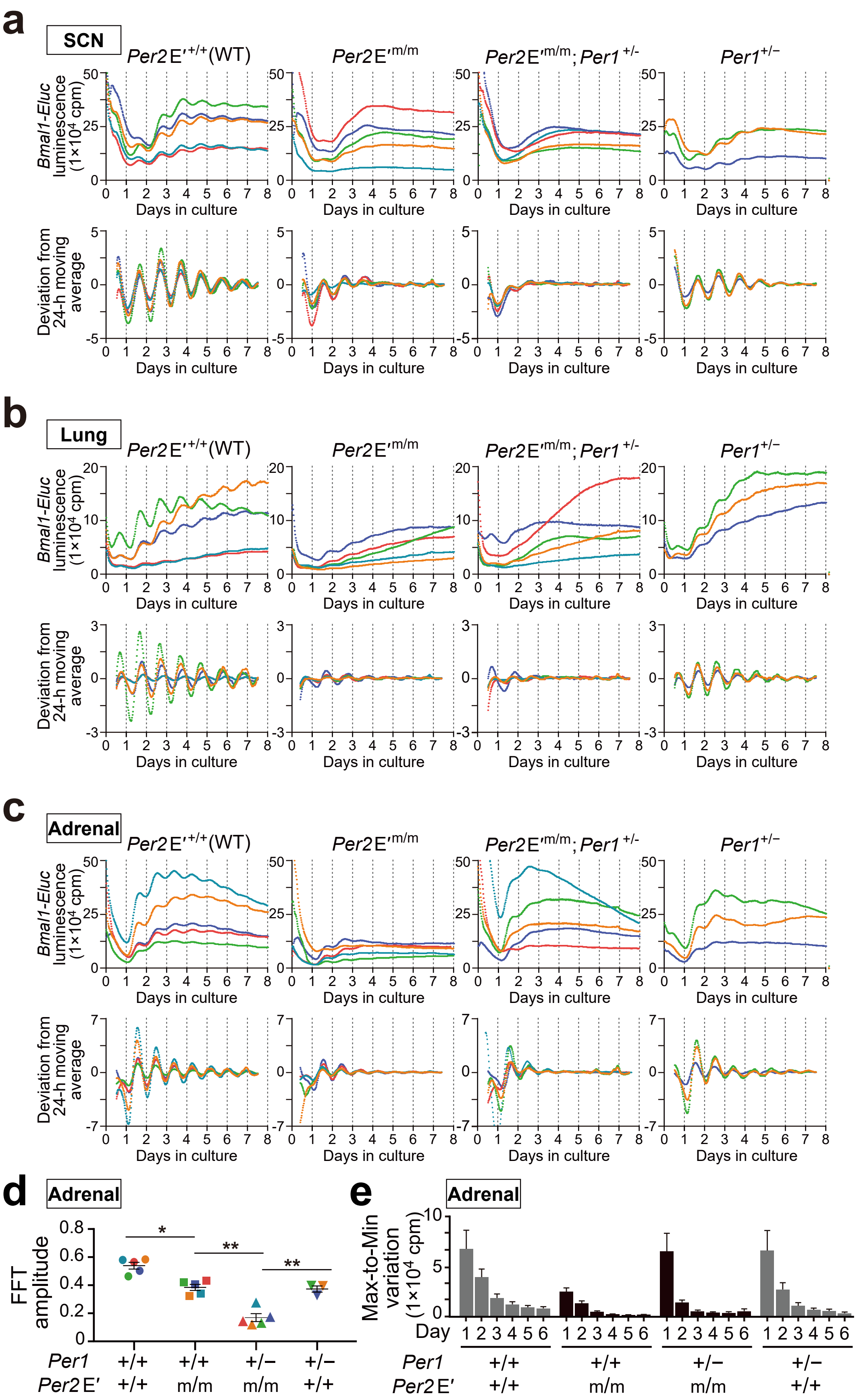

Supplementary Figure 4 (related to Fig. 3) | *Bmal1-Eluc* bioluminescence traces from ex vivo SCN, lung, and adrenal cultures. Raw data (upper) and detrended data (lower) of the same sets of SCN (**a**), lung (**b**), and adrenal (**c**) cultures from *Per2*E′$^{+/+}$ (*n*=5), *Per2*E′$^{m/m}$ (*n*=5), *Per2*E′$^{m/m}$;*Per1*$^{+/-}$ (*n*=5), and *Per1*$^{+/-}$ (*n*=3) mice are shown. The detrended data of the SCN and the lung slice cultures are reproduced form **Figs. 3a** and **3d**. (**d**) FFT amplitude of adrenal *Bmal1-Eluc* data in (**c**). *P<0.05, **P<0.001, one-way ANOVA, Bonferroni *post hoc* test. (**e**) Daily max-to-min variations of (**c**). The data are presented as the mean ± s.e.m. Source data are provided as a Source Data file.

Supplementary Figure 5

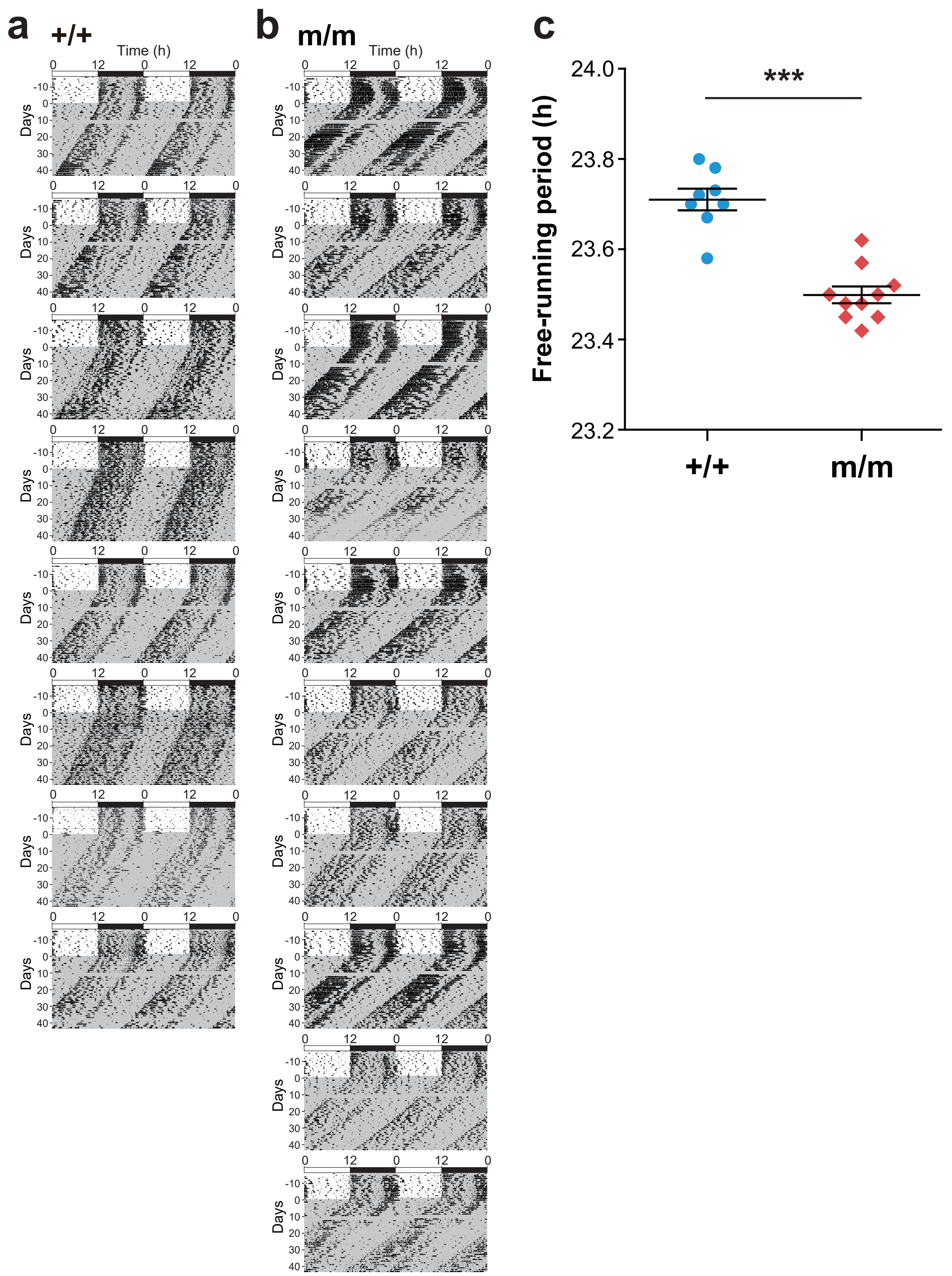

Supplementary Figure 5 | *Per2*E′$^{m/m}$ mice display a short free-running period of locomotor activity rhythms in DD. Double-plotted actograms of WT (**a**) and *Per2*E′$^{m/m}$ (**b**) mice. Animals were maintained in LD and then transferred to DD (days 0 to 44). At day 10, the recording was disrupted for about 45 hr. (**c**) Circadian periods of free-running activities in DD. The free-running period was estimated by χ^2 periodogram from days 4–43 in DD. Bars indicate mean period ± s.e.m. (WT, n = 8; *Per2*E′$^{m/m}$, n = 10). ***P<0.0001, Student's t-test.

Supplementary Figure 6

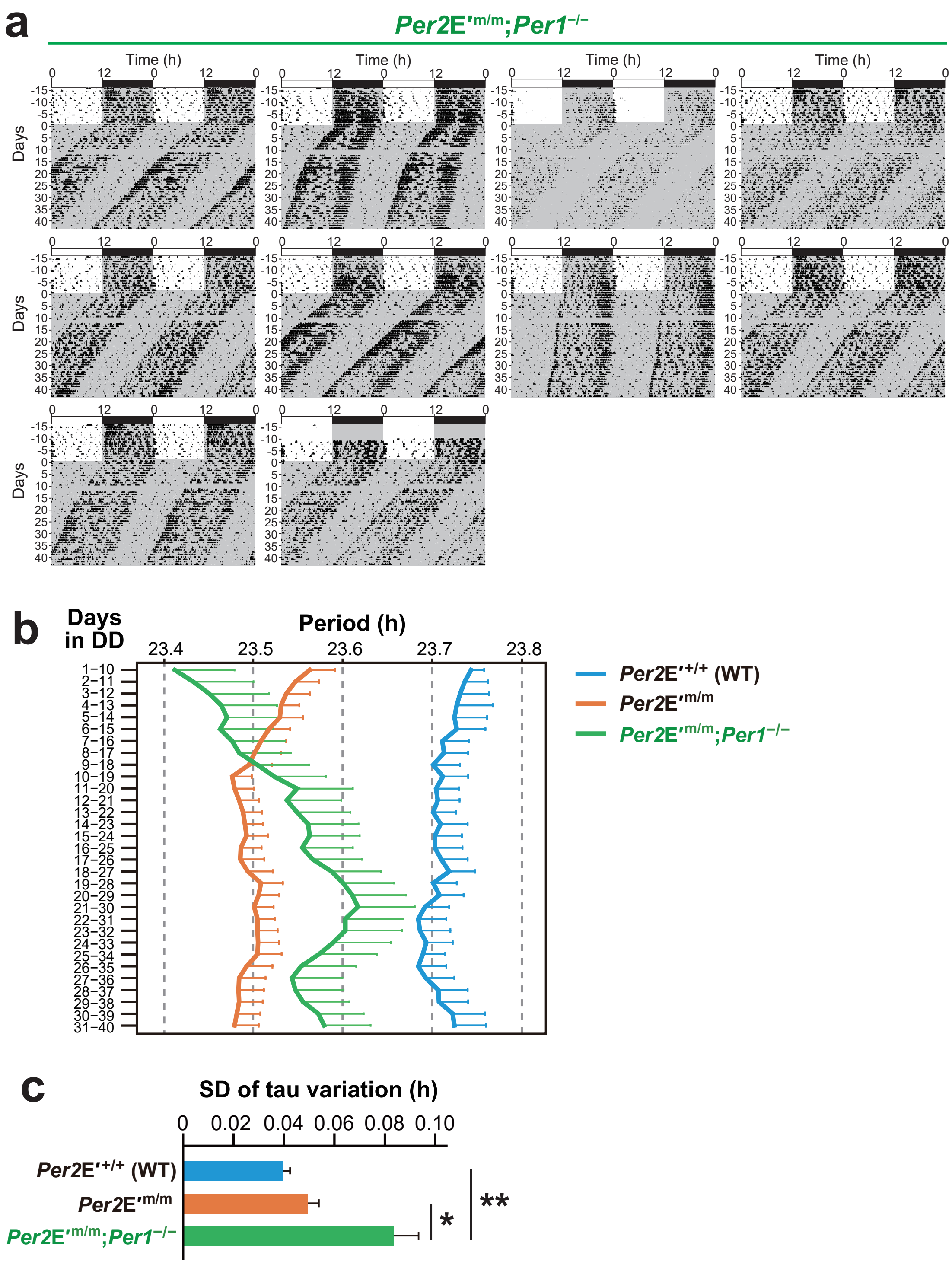

Supplementary Figure 6 | *Per2*E′$^{m/m}$;*Per1*$^{-/-}$ mice display an unstable free-running period of locomotor activity rhythms in DD. (**a**) Double-plotted actograms of *Per2*E′$^{m/m}$;*Per1*$^{-/-}$ mice. Animals were maintained in LD and then transferred to DD. At day 10, the recording was disrupted for about 45 hr. (**b**) Daily plots of tau at 10-day intervals in DD (mean ± s.e.m.) for *Per2*E′$^{+/+}$(WT) (n = 8), *Per2*E′$^{m/m}$ (n = 10), and *Per2*E′$^{m/m}$;*Per1*$^{-/-}$ (n = 10). Tau was calculated with χ^2 periodogram. (**c**) Standard deviation (SD) of tau variations for 40 days in (**b**). Data indicate mean ± s.e.m. *P<0.05, **P<0.01, one-way ANOVA, Bonferroni *post hoc* test. Source data for (**b**) and (**c**) are provided as a Source Data file.

Supplementary Figure 7

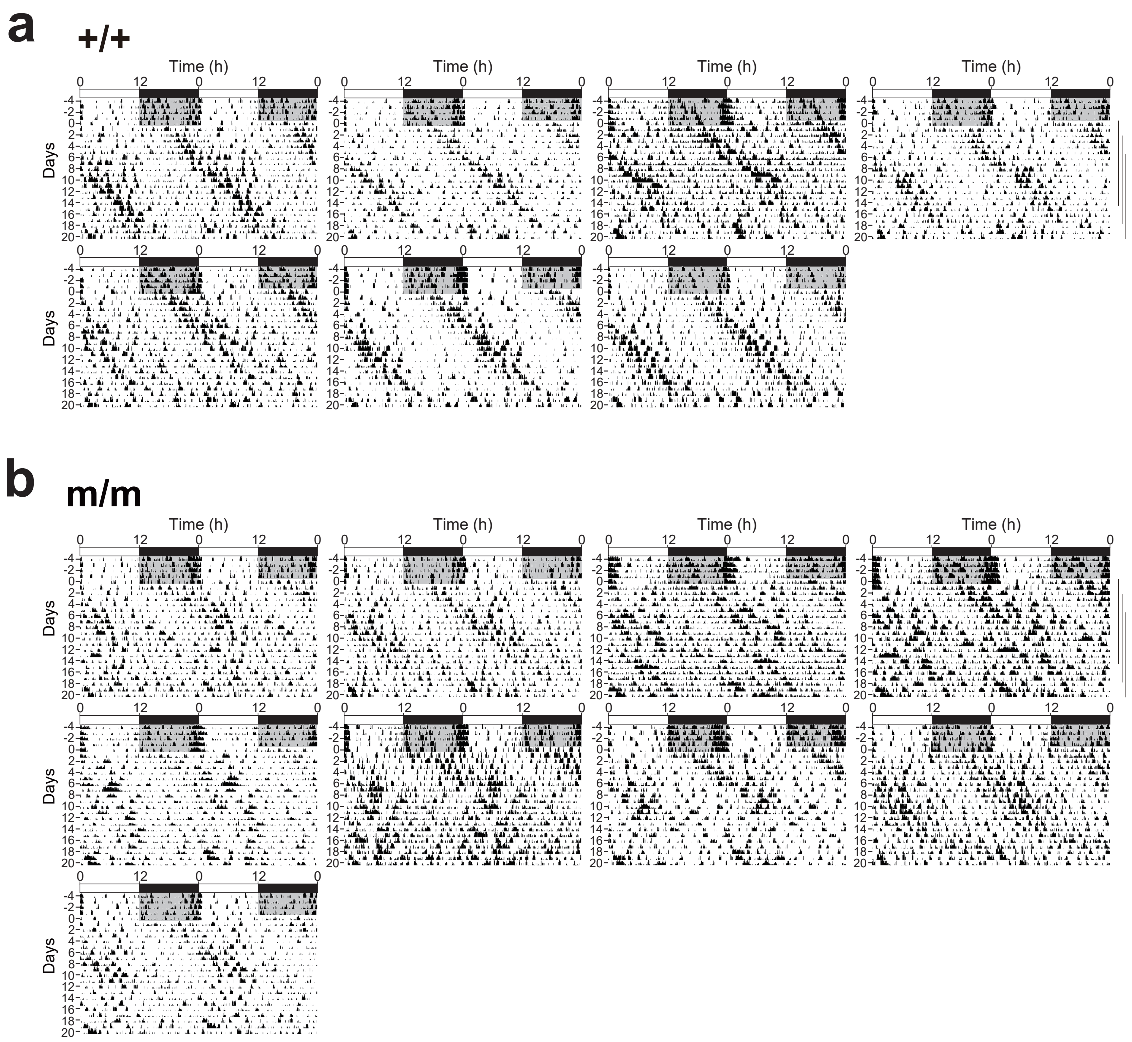

Supplementary Figure 7 (related to Fig. 4a) | Double-plotted actograms of WT (**a**) and *Per2*E′$^{m/m}$ (**b**) mice. Animals were maintained in LD and then transferred to LL.

Supplementary Figure 8

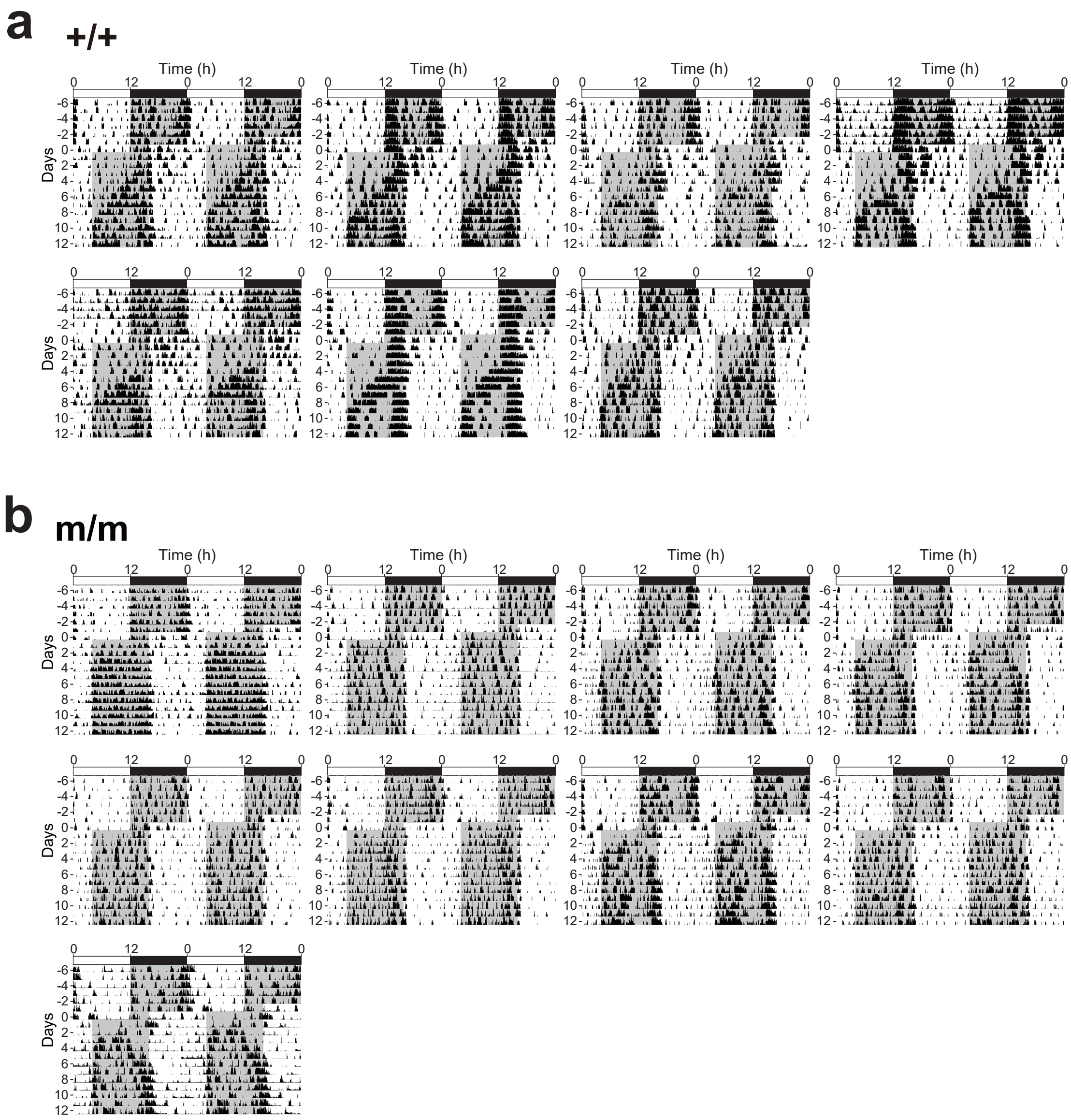

Supplementary Figure 8 (related to Fig. 4c) | Double-plotted actograms of WT (**a**) and *Per2*E′[m/m] (**b**) mice subjected to 8-hr phase advance in LD cycles.

Supplementary Figure 9

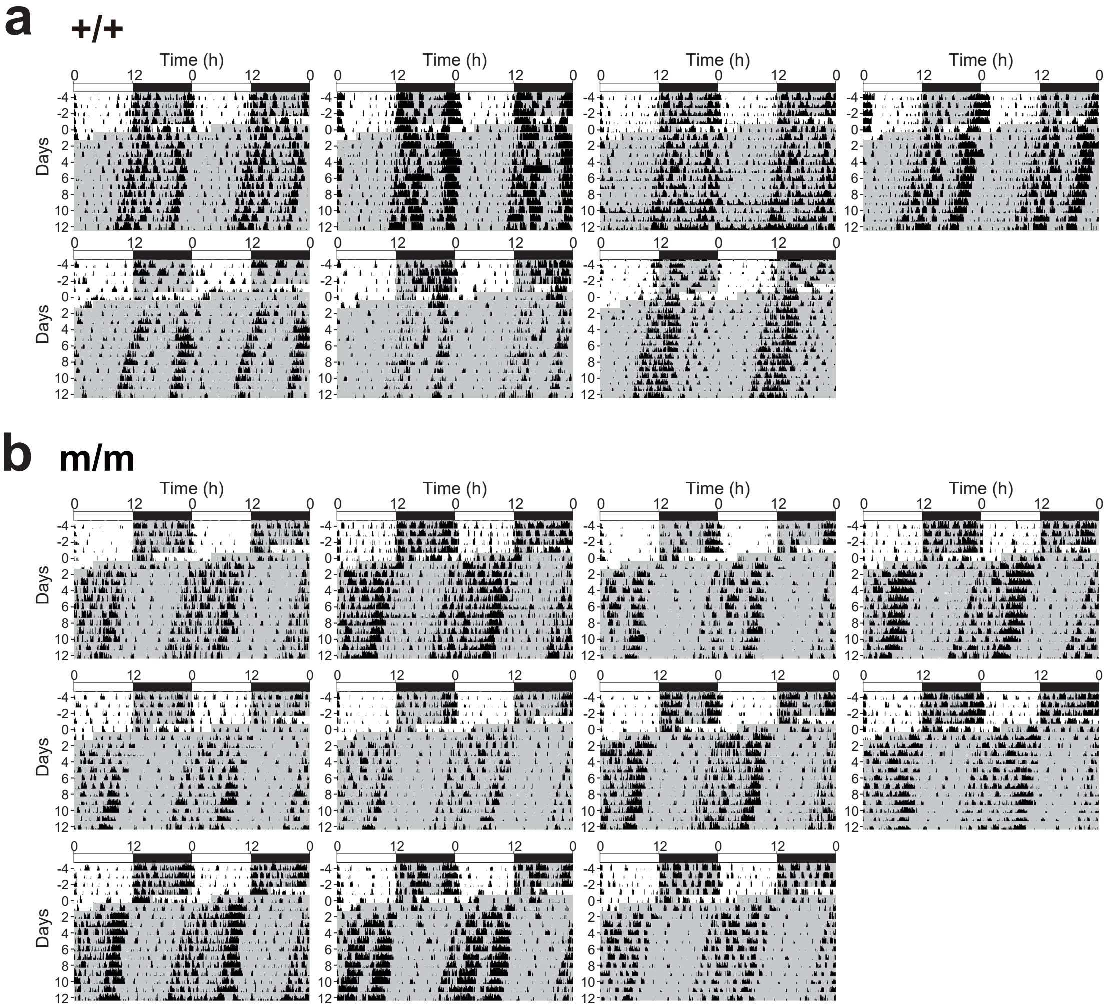

Supplementary Figure 9 (related to Fig. 4d) | Double-plotted actograms of WT (**a**) and *Per2*E$^{m/m}$ (**b**) mice. We advanced LD cycles by 8 hr transiently for 1 day and turned off the light.

Supplementary Figure 10

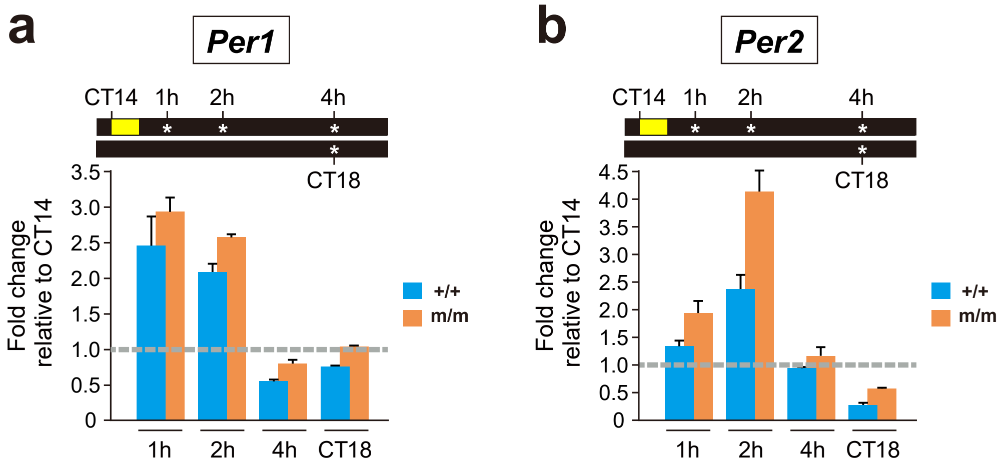

Supplementary Figure 10 | Expression profiles of *Per1* and *Per2* in the SCN of WT (+/+) and *Per2*E′$^{\mathbf{m/m}}$ (m/m) mice after a 30-min light pulse exposure at CT14. Mice were sacrificed 1, 2, 4 h after the light onset. No-light-exposed animals at CT18 served as control groups. The SCN was dissected out by laser microdissection, and mRNA levels of *Per1* (**a**) and *Per2* (**b**) were determined by qRT-PCR and normalized to those of the ribosomal phosphoprotein P0 (Rplp0)-encoding gene. Data are plotted as means ± variation (n = 2 mice for each data point), relative to basal values at CT14. Note that the *Per2*E′$^{m/m}$ mutation augments light-induced expression of *Per2* but not *Per1*. Source data are provided as a Source Data file.

Supplementary Figure 11

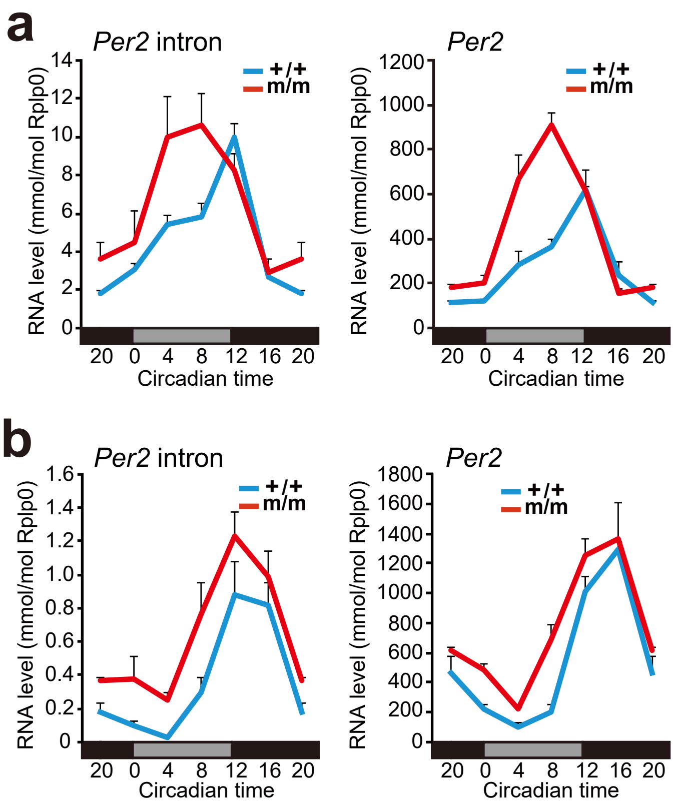

Supplementary Figure 11 | Altered circadian expression of *Per2* in the SCN and liver in the *Per2* E′-box mutant mice. Temporal profiles of *Per2* transcripts in the SCN (**a**) and the liver (**b**) of *Per2*E′$^{+/+}$ and *Per2*E′$^{m/m}$ mice are shown. The levels of *Per2* mRNA and pre-mRNA (intronic RNA) were determined by qRT-PCR and normalized to those of the ribosomal phosphoprotein P0 (Rplp0)-encoding gene. Data at CT20 are double-plotted. Values are the means ± s.e.m. (*n* = 3-5 mice for each data point for SCN, *n* = 2-3 mice for liver).
