# Supplementary material for: Non-coding cis-element of Period2 is essential for maintaining organismal circadian behaviour and body temperature rhythmicity
Source: Nat Commun. 2019 Jun 12;10:2563. doi: 10.1038/s41467-019-10532-2 (PMC6561950; doi:10.1038/s41467-019-10532-2)
Supplement: Supplementary file 3 — Reporting Summary [file 41467_2019_10532_MOESM3_ESM.pdf]

nature research

Corresponding author(s): Masao Doi and Hitoshi Okamura

Last updated by author(s): Apr 28, 2019

Reporting Summary

Nature Research wishes to improve the reproducibility of the work that we publish. This form provides structure for consistency and transparency in reporting. For further information on Nature Research policies, see Authors & Referees and the Editorial Policy Checklist.

Statistics

For all statistical analyses, confirm that the following items are present in the figure legend, table legend, main text, or Methods section.

n/a	Confirmed	
☐	☒	The exact sample size (n) for each experimental group/condition, given as a discrete number and unit of measurement
☐	☒	A statement on whether measurements were taken from distinct samples or whether the same sample was measured repeatedly
☐	☒	The statistical test(s) used AND whether they are one- or two-sided<br>*Only common tests should be described solely by name; describe more complex techniques in the Methods section.*
☒	☐	A description of all covariates tested
☒	☐	A description of any assumptions or corrections, such as tests of normality and adjustment for multiple comparisons
☐	☒	A full description of the statistical parameters including central tendency (e.g. means) or other basic estimates (e.g. regression coefficient) AND variation (e.g. standard deviation) or associated estimates of uncertainty (e.g. confidence intervals)
☒	☐	For null hypothesis testing, the test statistic (e.g. F, t, r) with confidence intervals, effect sizes, degrees of freedom and P value noted<br>*Give P values as exact values whenever suitable.*
☒	☐	For Bayesian analysis, information on the choice of priors and Markov chain Monte Carlo settings
☒	☐	For hierarchical and complex designs, identification of the appropriate level for tests and full reporting of outcomes
☒	☐	Estimates of effect sizes (e.g. Cohen's d, Pearson's r), indicating how they were calculated

Our web collection on statistics for biologists contains articles on many of the points above.

Software and code

Policy information about availability of computer code

Data collection	LAS4000 mini 1.0 (Fuji film) for western blot analysis.<br>StepOne 2.2.2 (Thermo Fisher) for realtime PCR analysis.<br>Kronos 2.30 (ATTO) for bioluminescence recording.<br>CLOCKLAB (Actimetrics) for circadian locomotor activity analysis.<br>OneWireViewer 0.3.19.47 (Maxim Integrated) for body temperature recording.
Data analysis	Multi Gauge 3.1(Fuji film) for western blot analysis.<br>Excel (Microsoft) for FFT analysis.<br>CLOCKLAB (Actimetrics) for circadian locomotor activity analysis.<br>Oriana 4 (Kovacs Computer Services) for circular statistics.<br>Excel (Microsoft) and Prism 8.0 (GraphPad) for statistical analysis.

For manuscripts utilizing custom algorithms or software that are central to the research but not yet described in published literature, software must be made available to editors/reviewers. We strongly encourage code deposition in a community repository (e.g. GitHub). See the Nature Research guidelines for submitting code & software for further information.

Data

Policy information about availability of data

All manuscripts must include a data availability statement. This statement should provide the following information, where applicable:

- Accession codes, unique identifiers, or web links for publicly available datasets
- A list of figures that have associated raw data
- A description of any restrictions on data availability

The authors declare that the data supporting the findings of this study are available within the article and its Supplementary Information files. The source data underlying Figs. 1c,e, 2a,b, 3c,f, 4a,b,c,d, Supplementary Figures 3, 4e, 6b,c, 10a,b, 11a,b are provided as a Source Data file.

Field-specific reporting

Please select the one below that is the best fit for your research. If you are not sure, read the appropriate sections before making your selection.

☒ Life sciences ☐ Behavioural & social sciences ☐ Ecological, evolutionary & environmental sciences

For a reference copy of the document with all sections, see nature.com/documents/nr-reporting-summary-flat.pdf

Life sciences study design

All studies must disclose on these points even when the disclosure is negative.

Sample size	No statistical method was used to predetermine sample size.
Data exclusions	No data were excluded.
Replication	Except for ChIP assay, for which we used technical replicates, all experiments were performed using biological replicates. The number of experiments is described in the figure legends.
Randomization	No randomization was applied in this study.
Blinding	The investigators were not blinded to the samples.

Reporting for specific materials, systems and methods

We require information from authors about some types of materials, experimental systems and methods used in many studies. Here, indicate whether each material, system or method listed is relevant to your study. If you are not sure if a list item applies to your research, read the appropriate section before selecting a response.

Materials & experimental systems

n/a	Involved in the study
☐	☒ Antibodies
☐	☒ Eukaryotic cell lines
☒	☐ Palaeontology
☐	☒ Animals and other organisms
☒	☐ Human research participants
☒	☐ Clinical data

Methods

n/a	Involved in the study
☒	☐ ChIP-seq
☒	☐ Flow cytometry
☒	☐ MRI-based neuroimaging

Antibodies

Antibodies used	anti-mPER2 affinity-purified rabbit polyclonal antibody (Chronobiol Int 35, 132-136, 2018)<br>anti-mPER1 rabbit antiserum (Millipore, #AB2201)<br>anti-mCRY1 affinity-purified guinea pig polyclonal antibody (Mol Cell Biol 24, 584-594, 2004)<br>anti-mCRY2 affinity-purified guinea pig polyclonal antibody (Mol Cell Biol 24, 584-594, 2004)<br>anti-mCLOCK mouse monoclonal antibody (MBL, #D349-3)<br>β-Actin antibody (Sigma, A5441)
Validation	The specificity was validated on the manufacturer's website and literature (Chronobiol Int 35, 132-136, 2018; Millipore, #AB2201; Mol Cell Biol 24, 584-594, 2004; MBL, #D349-3; Sigma, A5441)

Eukaryotic cell lines

Policy information about cell lines

Cell line source(s)	Primary fibroblasts from WT (Per2E'+/+) and mutant (Per2E'm/m) mouse lung tissues
Authentication	No authentication was performed.
Mycoplasma contamination	The cells were not tested for mycoplasma contamination.
Commonly misidentified lines<br>(See ICLAC register)	No commonly misidentified lines were used.

Animals and other organisms

Policy information about studies involving animals; ARRIVE guidelines recommended for reporting animal research

Laboratory animals	Wild-type mice, C57BL/6J<br>Per2 E'-box mutant mice, C57BL/6J<br>Bmal1-Eluc reporter transgenic mice, C57BL/6J<br>Per1-deficient mice, C57BL/6J
Wild animals	This study did not involve wild animals.
Field-collected samples	This study did not involve samples collected from the field.
Ethics oversight	All animal experiments were performed under protocols approved by the Animal Care and Experimentation Committee of Kyoto University and Institutional Animal Care and Use Committee of RIKEN Kobe Branch.

Note that full information on the approval of the study protocol must also be provided in the manuscript.
